# Complete chloroplast genome sequence of the Tibetan catnip *Nepeta hemsleyana* Oliver ex Prain (Lamiaceae)

**DOI:** 10.1080/23802359.2022.2054737

**Published:** 2022-03-28

**Authors:** Mary Ann C. Bautista, Xuemei Wen, Chao Ma, Yanli Tu, Tao Chen

**Affiliations:** a Shenzhen Fairy Lake Botanical Garden, Chinese Academy of Sciences, Shenzhen, China; b International School, University of Chinese Academy of Sciences, Beijing, China; c Tibet Plateau Institute of Biology, Lhasa, China

**Keywords:** Chloroplast genome, Lamiaceae, Mentheae, *Nepeta hemsleyana*, phylogeny

## Abstract

*Nepeta hemsleyana* Oliver ex Prain (1891) is one of the aromatic Tibetan herbs used to treat convulsions. In this study, the complete chloroplast genome of *N. hemsleyana* was analyzed and is presented here for the first time. The assembled genome, 152,171 bp in length, contained a large single-copy region (82,214 bp) and a small single-copy region (17,605 bp) separated by a pair of inverted repeats (25,676 bp). A total of 131 genes were identified, including 86 protein-coding genes, 37 transfer RNA genes, and eight ribosomal RNA genes. The phylogenetic analysis also confirmed the early divergence of *N. hemsleyana* from other species in subtribe Nepetinae.

Species of *Nepeta* L. (Lamiaceae) are herbaceous perennials known for their aromatic foliage and flowers (Li and Hedge 1994; Jamzad et al. 2003). With nearly 300 species, *Nepeta* is one of the largest genera in tribe Mentheae, subfamily Nepetoideae (Jamzad et al. 2003). Species of *Nepeta* are considered to be a rich source of compounds with numerous pharmacological effects; however, species delimitation among this group remains unclear. The morphological characters of *Nepeta* are highly variable, even within closely related species, and relationships with allied genera need further investigation (Jamzad et al. 2003). In line with this, we sequenced the complete chloroplast genome of *Nepeta hemsleyana* Oliver ex Prain, a medicinally important species of grassy slopes around Lhasa, Tibet. The information obtained from the chloroplast genome can contribute to future studies that attempt to resolve taxonomic relationships among the species of *Nepeta* and their close allies.

Total genomic DNA was extracted from fresh leaves of the *Nepeta hemsleyana* plant using the modified CTAB method (Murray and Thompson 1980). The material was collected in Chengguan, Lhasa, Tibet, China (E 91°9′50″, N 29°46′10″). A voucher specimen (2020072401) was deposited in the herbarium of the Tibet Plateau Institute of Biology (XZ, Tu Yanli, sws_wenxuemei@sti.xizang.gov.cn). Short insert-sized libraries (350 bp) were constructed using the manufacturer’s protocol for the Nextera XT DNA Library Preparation Kit. Sequencing was carried out utilizing the Illumina Novaseq 600 platform. Sequencing resulted in 150 bp paired-end reads with an average sequencing depth of 2824.9 X. After filtering the raw reads, the chloroplast genome was assembled using the *de novo* assembler SPAdes 3.11.0 (Vasilinetc et al. 2015). The assembled plastome was annotated using Plann software (Huang and Cronk 2015), RNAmmer 1.2 (Lagesen et al. 2007), and tRNAscan-SE (Chan and Lowe 2019) for protein-coding genes, rRNA genes, and tRNA genes, respectively.

**Figure 1.**
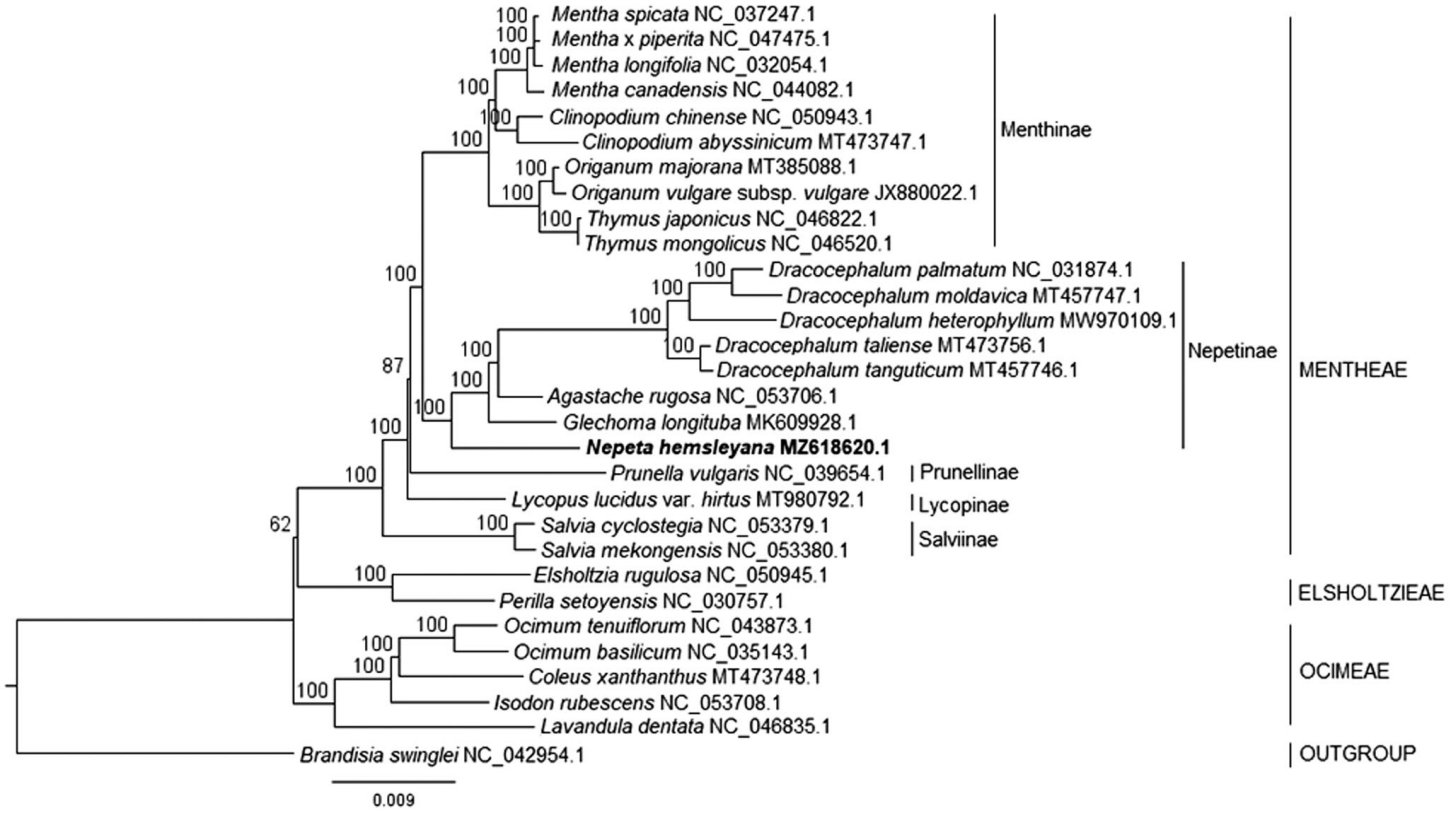
Maximum-likelihood consensus tree showing phylogenetic placement of *Nepeta hemsleyana* within tribe Mentheae. Bootstrap support values based on 1000 iterations are indicated above the nodes; GenBank accession numbers follow the binomials.

The complete chloroplast genome of *Nepeta hemsleyana* (MZ618620) was 152,171 bp in length and displayed a typical quadripartite structure. It contained a large single-copy (LSC) region (82,214 bp), a small single-copy (SSC) region (17,605 bp), and a pair of inverted repeats (25,676 bp). The annotated genome had 131 genes, comprising 86 protein-coding genes, 37 tRNA genes, and eight rRNA genes. Six protein-coding genes, seven tRNA genes, and four rRNA genes were duplicated in the IR regions. Among the identified genes, 15 genes (*trn*K-UUU, *rps*16, *trn*G-UCC, *atp*F, *rpo*C1, *trn*L-UAA, *trn*V-UAC, *pet*B, *pet*D, *rpl*16, *rp*l2, *ndh*B, *trn*I-GAU, *trn*A-UGC, *ndh*A) had a single intron, while *clp*P and *ycf*3 had two introns each; *rps*12 was also a trans-spliced gene. Overall, the GC content was 37.9% and highly similar to other sequences from the tribe Mentheae. Moreover, the GC content of the IR regions (43%) was higher than the LSC (35.9%) and SSC (31.9%) regions.

To determine the phylogenetic position of *Nepeta hemsleyana* within the tribe Mentheae and subfamily Nepetoideae, 28 chloroplast genome sequences from the same subfamily were used for phylogenetic reconstruction (Figure 1). Maximum-likelihood (ML) analysis with the GTR + I+G nucleotide substitution model was conducted in RAxML 8.2.11 (Stamatakis 2014). *Brandisia swinglei* was designated as the outgroup. The ML analysis revealed that *N. hemsleyana* belonged to the Nepetinae clade and was basal to other genera of Nepetinae, such as *Glechoma*, *Agastache*, and *Dracocephalum*. The tree also showed that subtribe Nepetinae had a sister-group relationship with subtribe Menthinae.

## Data Availability

The complete chloroplast genome sequence of *Nepeta hemsleyana* was deposited in the NCBI GenBank database (https://www.ncbi.nlm.nih.gov/nuccore/MZ618620) under accession number MZ618620. Raw sequencing data are available in the SRA database (https://www.ncbi.nlm.nih.gov/sra/SRX11580391[accn]) with the BioSample number SAMN20446770, SRA number SRX11580391, and BioProject accession number PRJNA672143.
